# Risk factors and drug resistance of non-tuberculous mycobacteria in HIV/AIDS patients: a retrospective study in southern China

**DOI:** 10.3389/fpubh.2025.1660472

**Published:** 2025-09-26

**Authors:** Jing Ye, Qingpeng Yang, Yan Huang, Mei Lin, Xiaomin Xian, Liwen Huang, Huifang Qin, Chongxing Zhou, Yingkun Zhang, Xiaoyan Liang, Jin Ou, Zhezhe Cui

**Affiliations:** ^1^Guangxi Key Laboratory of Major Infectious Disease Prevention and Control and Biosafety Emergency Response, Guangxi Key Discipline Platform of Tuberculosis Control, Guangxi Centre for Disease Control and Prevention, Nanning, China; ^2^School of Public Health, The Key Laboratory of Environmental Pollution Monitoring and Disease Control, Ministry of Education, Guizhou Medical University, Guiyang, China

**Keywords:** non-tuberculous mycobacteria (NTM), HIV/AIDS, interferon-γ release assay, drug resistance, gene chip

## Abstract

**Background:**

The incidence and infection rate of *Non-tuberculous Mycobacteria* (NTM) are increasing across different regions, with regional variations in the types, distribution, and drug resistance profiles. Our objective was to investigate the risk factors, distribution of predominant Mycobacteria species, and phenotypic drug resistance profiles in co-infected HIV/AIDS patients in southern China.

**Methods:**

Blood and sputum samples were collected from 2,985 HIV/AIDS patients without prior history of pulmonary tuberculosis (PTB) in five designated hospitals in Guangxi, southern China from January 2019 to December 2020. Univariate analysis and binary logistic regression models were used to explore the related risk factors of HIV/AIDS patients with NTM infection and those with *Mycobacterium tuberculosis* (MTB) infection, respectively. Interferon-γ release assay (IGRA) tests and CD4+ counts were performed on blood samples, Roche medium was used for sputum culture, and positive isolates underwent species identification and drug susceptibility testing.

**Results:**

*Mycobacterium tuberculosis* and NTM culture positivity rates were 1.2% (35/2985) and 2.2% (66/2985), respectively (*χ*^2^ = 9.679, *p* = 0.002). Predominant NTM pathogens were *Mycobacterium avium* (28.8%, 19/66), *Mycobacterium fortuitum* (21.2%, 14/66), and *Mycobacterium chelonae/abscessus complex* (16.7%, 11/66). Multivariate analysis revealed cough (Adj. OR: 192.47, 95%*CI*: 15.71–2357.63, *p* < 0.001) and farming (Adj. OR: 20.92, 95%*CI*: 1.33–328.93, *p* = 0.031) as risk factors for NTM co-infection, whereas other pulmonary symptoms increased risk of MTB infection (Adj. OR: 3.37, 95% *CI*: 1.03–11.08, *p* = 0.045). Cough significantly differed between NTM and MTB groups (*χ*^2^ = 66.070, *p* < 0.001). Sixty-six NTM strains were tested for resistance to 10 common antibiotics. The drug resistance rates of para-aminosalicylic acid (PAS), Isoniazid (INH), Levofloxacin (LFX), Kanamycin (K), Ethambutol (EMB), Capreomycin (CPM), Rifampin (RFP), Moxifloxacin (MFX) and Amikacin (AM) exceeded 50.0%., while Protionamide (TH1321) was 25.8%. There was no significant in interferon status distribution across CD4+ counts groups (*p* = 0.574).

**Conclusion:**

For HIV/AIDS patients presenting with cough symptoms, it is recommended that molecular biology techniques be employed concurrently with MTB testing to screen for and identify NTM, thereby clarifying the specific type of mycobacterial infection present. IGRA cannot completely distinguish MTB from NTM, and more auxiliary examinations are needed.

## 1 Introduction

NTM refers to a group of mycobacteria except MTB and *Mycobacterium leprosy* (1, 2). In recent years, the incidence and infection rate of NTM have gradually increased in different regions of the world, which has become a new public health problem (3, 4). Moreover, the types, distribution, and drug resistance profiles of NTM strains differ across regions, reflecting regional variations (4–6). NTM is widely found in the environment and can cause invasion of human lung, lymph node and central nervous system and pulmonary infection is more common. Previous studies have shown that NTM are opportunistic pathogens that are more likely to cause infections in people with weakened immune systems (7–9). In the past decade, the incidence of AIDS in China has been on the rise in most areas, and the southwest area was the high incidence area, among which Guangxi region had become the “high-high” cluster area of AIDS earlier (10). The overall infection rate of the population in Liuzhou was higher than the national infection rate of human immunodeficiency virus (HIV) patients in 2022, and the AIDS epidemic in Liuzhou was still at a high risk (11). The prevalence of HIV in Guangxi region has to some extent facilitated the spread of NTM infections (12, 13). NTM is also a significant contributor to pulmonary and disseminated infections, as well as associated mortalities (14, 15). NTM lung disease is very similar to PTB in clinical manifestations, bacterial morphology and imaging manifestations. If the bacteria are not identified in time, it is easy to be mistaken for tuberculosis, leading to treatment failure (16). In China, tuberculosis patients are mainly identified passively when they present with suspicious symptoms of pulmonary tuberculosis (such as coughing, expectoration, hemoptysis, and chest pain) or during the treatment for other diseases (17). Therefore, when screening for tuberculosis among HIV/AIDS patients, the screening is mainly conducted on individuals who have already shown symptoms related to tuberculosis. There is no screening for asymptomatic PTB in HIV/AIDS patients, but they are already infectious (18, 19).

In order to further understand the prevalence and drug resistance of NTM in HIV/AIDS patients in Guangxi, it is of great significance for formulating therapeutic regimen and prevention strategies. Therefore, the samples of HIV/AIDS patients without prior history of PTB from 2019 to 2020 were collected for strain identification and drug sensitivity test.

## 2 Methods

### 2.1 Data and specimen sources

**Figure 1:**
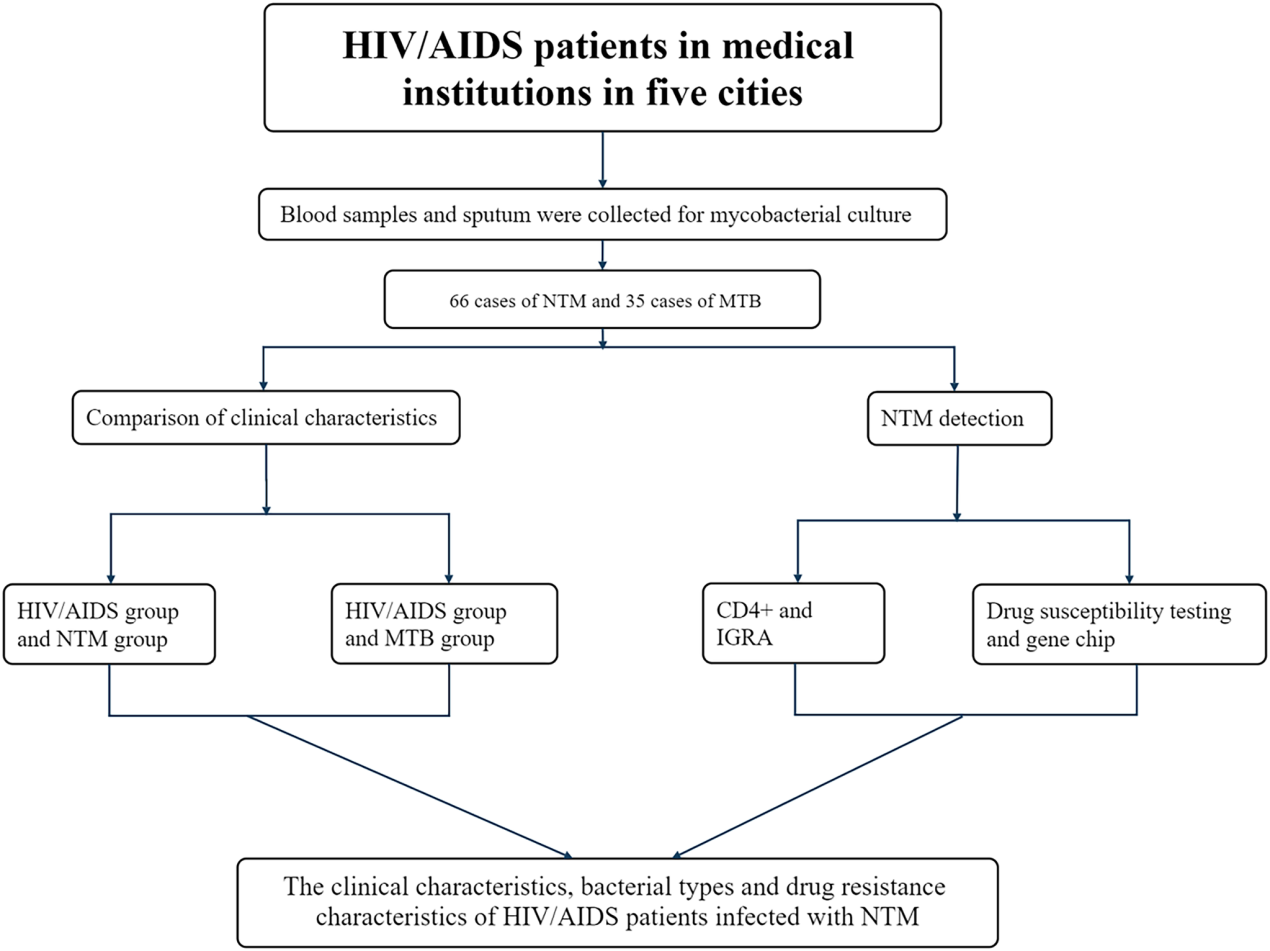
The schematic diagram of the design workflow for this study.

This study adopted a retrospective observational research design. Stratified by geographical attributes, five cities were randomly selected from the east, west, south, north and center in Guangxi region of southern China as monitoring points (Nanning, Liuzhou, Guigang, Laibin, and Chongzuo). From 2019 to 2020, 2,985 blood and sputum samples were collected from HIV/AIDS patients without prior PTB history at designated medical institutions in these cities. Clinical characteristics of the patients were also recorded using structured questionnaires (Figure 1).

### 2.2 Detection methods

For the application of the index detection methods—including the IGRA, CD4 + T lymphocyte counting, mycobacterial culture, and gene chip identification—we strictly followed the protocols provided by the reagent manufacturers. Detailed procedural descriptions of these detection methods were provided in the “Supplementary Material - Detection Methods”.

#### 2.2.1 NTM drug resistance detection

Phenotypic detection was performed by a traditional solid drug sensitivity test. NTM strains were prepared as a 1 mg/mL bacterial suspension, diluted to 10^−2^ mg/mL and 10^−4^ mg/mL, and inoculated onto control and drug-containing media using the streak-line method. Cultures were incubated at 37 °C for 4 weeks, after which results were recorded. The drug culture medium (Zhuhai BASO Biotechnology Co., Ltd.) was used for the test. The tested drugs and concentrations were 2 μg/mL EMB, 30 μg/mL K, 1 μg/mL CPM, 1 μg/mL PAS, 0.2 μg/mL INH, 40 μg/mL RFP, 30 μg/mL AM, 1 μg/mL TH1321, 4 μg/mL LFX and 1 μg/mL MFX, totaling 10 kinds.

### 2.3 Statistical methods

R 4.4.3 statistical software was used to sort out and analyze the data. Mice package was used for multiple imputation of missing data. The count data were expressed as “n or %,” and the measurement data were expressed as X±S according to the normal distribution. Skewed distribution was expressed as “median (quartile) [*M* (*P_25_*, *P_75_*)].” Chi-square test, Fisher’s exact test and rank sum test were used to compare the difference of count data between groups. As the control group (HIV/AIDS without NTM/MTB) of the NTM co-infection group and the MTB co-infection group, the Propensity Score Matching (PSM) method was employed to balance the case group and the control group, in order to reduce the influence of confounding factors. The respective HIV/AIDS control groups were matched with 1:2 ratio score matching by gender (exact matching) and age (nearest neighbor matching) using R4.4.3 software. Univariate analysis and binary conditional logistic regression were used to analyze the related risk factors. All tests were two-sided probability tests, and *p <* 0.05 was considered statistically significant.

### 2.4 Ethics statement

The study protocol was approved by the review board of Guangxi Center for Disease Control and Prevention (GXCDCIRB 2024–0028). The study was executed by the ethical standards set in the 1964 Declaration of Helsinki and its later modifications. Because of the retrospective nature of this observational study, the requirement for written consent was waived. Clinical trial number: not applicable.

## 3 Results

### 3.1 Distribution of isolated NTM strains

**Figure 2:**
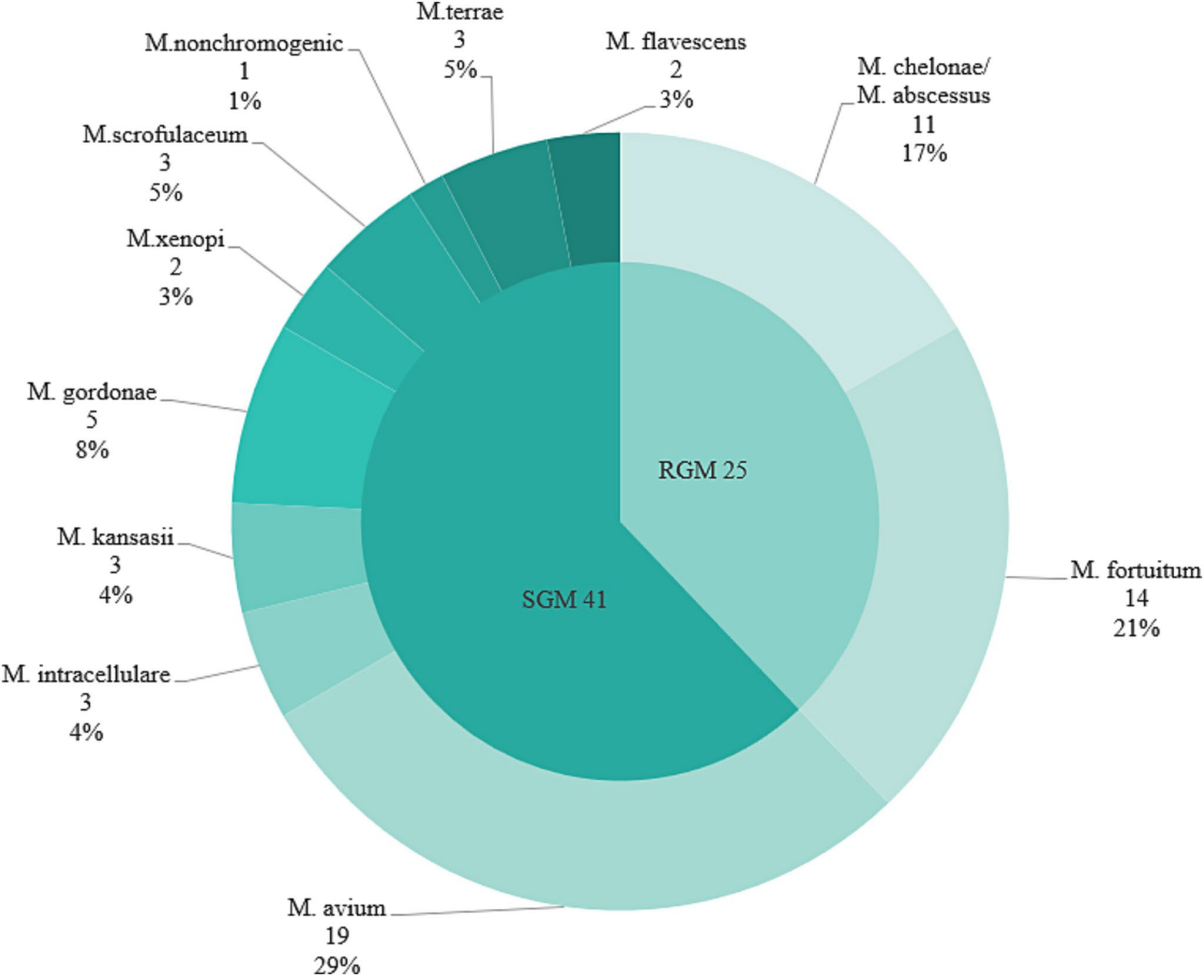
Proportion of epidemic strains of NTM in five urban districts in Guangxi region of southern China, 2019–2020. SGM, slow-growing mycobacteria; RGM, rapidly-growing mycobacteria.

Among 2,985 HIV/AIDS patients, the mycobacterial culture positive rate was 3.5% (104/2985). Of these, MTB culture positivity was 1.8% (35/2985), and NTM culture positivity was 2.2% (66/2985), the difference was statistically significant (*χ*^2^ = 9.679, *p* = 0.002). A total of 11 species of NTM were identified, including 41 slow-growing mycobacteria (SGM) and 25 rapidly-growing mycobacteria (RGM):19 *Mycobacterium avium* (*M. avium*), 14 *Mycobacterium fortuitum (M. fortuitum)*, 11 *Mycobacterium chelonae/abscessus complex* (*M. chelonae/abscessus complex*), 5 *Mycobacterium gordonae* (*M. gordonae*), 3 *Mycobacterium intracellulare* (*M. intracellulare*), 3 *Mycobacterium kansasii* (*M. kansasii*), 3 *Mycobacterium scrofulaceum* (*M. scrofulaceum*), 3 *Mycobacterium terrae* (*M. terrae*), 2 *Mycobacterium xenopi* (*M. xenopi*), 2 *Mycobacterium flavescens* (*M. flavescens*) and 1 *nonchromogenic mycobacteria* (*M. nonchromogenic*). The most prevalent pathogens were the *M. avium* (28.8%, 19/66), the *M. fortuitum* (21.2%, 14/66), and *M. chelonae/abscessus complex* (16.7%, 11/66) (Figure 2).

### 3.2 Analysis of demographic and clinical characteristics of HIV/AIDS group and co-infected with NTM group

Univariate analysis showed that most NTM patients had cough symptoms (60/66), significantly higher than the HIV/AIDS group (*p* < 0.001). Most patients in both groups were farmers with a significant difference between groups (*p* = 0.004). Chest imaging results indicated that most patients in both groups had normal findings, followed by pulmonary infection (*p* = 0.033). A significant difference was observed in underlying diseases between the two groups (*p* = 0.008). However, there was no significant difference in CD4^+^ lymphocyte count, number of cigarettes smoked per week, frequency of drinking alcohol per week, ethnicity, marriage and education (*p* > 0.05). Refer to Table 1 for detailed information and variable assignment was shown in Table 2.

**Table 1:** Analysis of demographic and clinical characteristics of HIV/AIDS and NTM patients.

Variables	Levels	Groups		*χ* ^2^	*p*-value
		HIV/AIDS (*n* = 132)	NTM (*n* = 66)		
CD4^+^T lymphocyte count	≥200	98 (74.2)	43 (65.1)	1.358	0.244
	<200	34 (25.8)	23 (34.9)		
Number of cigarettes smoked per week		0 (0,6)	0 (0,3)		0.986^a^
Frequency of drinking alcohol per week		0 (0,0)	0 (0,0)		0.464^a^
Ethnicity	Han	58 (46.8)	30 (45.5)	0.030	0.862
	Zhuang	66 (53.2)	36 (54.6)		
Marriage	Married	87 (65.9)	45 (68.2)	0.334	0.953
	Unmarried	18 (13.6)	9 (13.6)		
	Divorced	11 (8.3)	4 (6.1)		
	Widowed	16 (12.1)	8 (12.1)		
Education	Illiterate person	12 (9.1)	4 (6.1)		0.051^b^
	Elementary school	42 (31.8)	35 (53.0)		
	Secondary school	59 (44.7)	23 (34.9)		
	High school	16 (12.1)	3 (4.6)		
	University	3 (2.8)	1 (1.5)		
Occupation	Other*	33 (25.0)	5 (7.6)	13.070	0.004
	Housework and unemployment	30 (22.7)	11 (16.7)		
	Worker	10 (7.6)	4 (6.1)		
	Farmer	59 (44.7)	46 (69.7)		
Cough	No	115 (87.1)	6 (9.1)	109.469	<0.001
	Yes	17 (12.9)	60 (90.9)		
Results of chest imaging	Normal	75 (56.8)	50 (75.8)	6.827	0.033
	Pulmonary infection	34 (25.8)	9 (13.6)		
	Other**	23 (17.4)	7 (10.6)		
Underlying disease	No	102 (77.3)	61 (92.4)	9.941	0.008
	Yes	30 (22.7)	5 (7.6)		

Except for the number of cigarettes smoked per week and frequency of drinking alcohol per week were described by “median (quartile) [*M* (*P*_25_, *P*_75_)],” other data were described by “number of people (proportion/%) [*n* (%)]”; ^a^Wilcoxon rank sum test method; ^b^Fisher exact probability method.

Other*: Business services, officials and staff, driver, retirees and former employees, unspecified.

Other**: Lung lesion, pulmonary nodules, emphysema, tuberculosis, etc.

**Table 2:** Logistic regression analysis variable assignment table.

Variables	Assignment
Groups	HIV/AIDS = 0, NTM = 1
CD4^+^ lymphocyte	≥200 = 1, <200 = 2
Ethnicity	Han = 1, Zhuang = 2
Education	Illiterate person = 1, Elementary school = 2, Secondary school = 3, High school = 4, University = 5
Occupation	Other = 1, Housework and Unemployment = 2, Worker = 3, Farmer = 4
Marriage	Married = 1, Unmarried = 2, Divorced = 3, Widowed = 4
Cough	No = 1, Yes = 2
Results of chest imaging	Normal = 1, Pulmonary infection = 2, Other = 3
Underlying disease*	No = 1, Yes = 2

Underlying disease*: Pulmonary infection, pulmonary heart disease, hepatitis, diabetes, silicosis, chronic bronchitis, hypertension, coronary heart disease, tumor, mental illness, etc.

**Figure 3:**
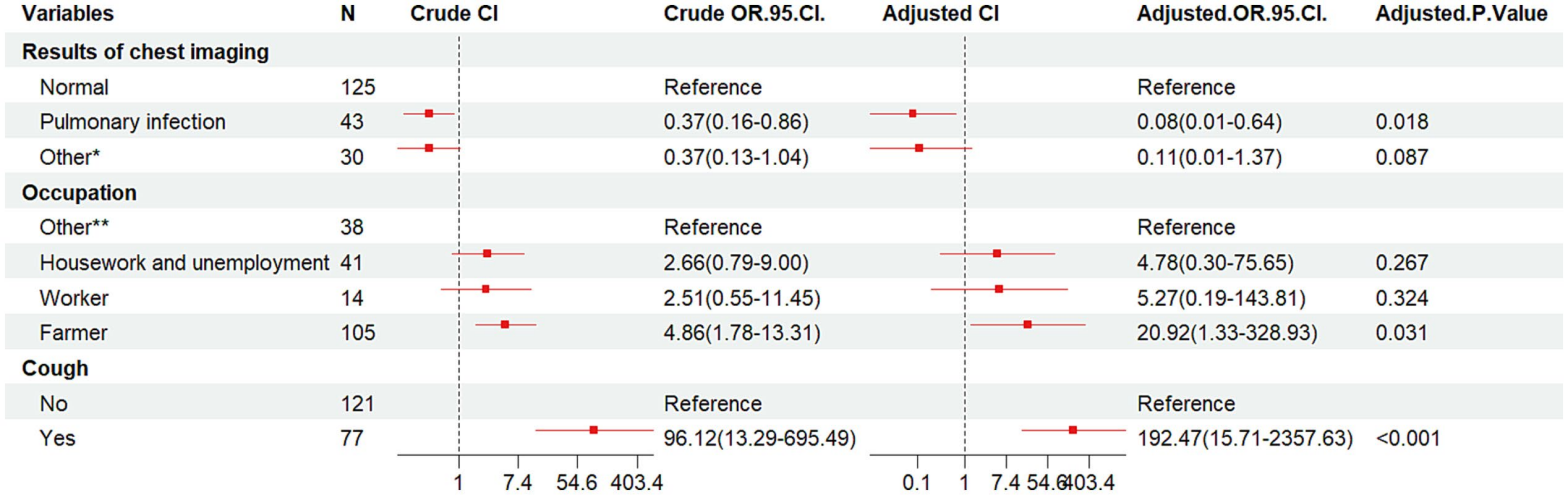
Forest plot of the results of conditional logistic regression analysis of pooled NTM infections. OR, odds ratio; CI, confidence interval; Other*, lung lesion, pulmonary nodules, emphysema, tuberculosis, etc.; Other**, business services, officials and staff, driver, non-executive and former employees, unspecified, etc.

Binary conditional logistic regression analysis was performed using HIV/AIDS and NTM co-infection as dependent variables, and 10 factors (CD4^+^T lymphocyte count, smoking, alcohol consumption, ethnicity, marriage, education, occupation, cough, chest imaging results, and underlying diseases) as independent variables (Table 3 and Figure 3). Results showed that cough (Crude OR: 96.12, 95%CI: 13.29–695.49; Adj. OR: 192.47, 95%CI: 15.71–2357.63) and farmer (Crude OR: 4.86, 95%CI: 1.78–13.31; Adj. OR: 20.92, 95%CI: 1.33–328.93) were risk factors for NTM co-infection. Compared to patients with normal chest imaging, those with pulmonary infection had a lower risk of NTM co-infection (Crude OR: 0.37, 95%CI: 0.16–0.86; Adj. OR: 0.08, 95%CI: 0.01–0.64).

**Table 3:** Multivariate logistic regression analysis of clinical characteristics of HIV/AIDS group and co-infected with NTM group.

Variables	Crude OR (95%CI)	Adj. OR (95%CI)	*P*-value (Wald’s test)	*P*-value (LR-test)
Results of chest imaging: Normal (reference)				0.013
Pulmonary infection	0.37 (0.16,0.86)	0.08 (0.01,0.64)	0.018	
Other*	0.37 (0.13,1.04)	0.11 (0.01,1.37)	0.087	
Occupation				0.039
Other** (reference)				
Housework and unemployment	2.66 (0.79,9.0)	4.78 (0.3,75.65)	0.267	
Worker	2.51 (0.55,11.45)	5.27 (0.19,143.81)	0.324	
Farmer	4.86 (1.78,13.31)	20.92 (1.33,328.93)	0.031	
Cough				<0.001
No (reference)				
Yes	96.12 (13.29, 695.49)	192.47 (15.71,2357.63)	<0.001	

OR, odds ratio; CI, confidence interval.

Other*: Lung lesion, pulmonary nodules, emphysema, tuberculosis, etc.

Other**: Business services, officials and staff, driver, retirees and former employees, unspecified, etc.

### 3.3 Analysis of demographic and clinical characteristics of HIV/AIDS group and co-infected with MTB group

Univariate analysis showed that the majority of patients in both groups were farmers by occupation with no statistically significant difference between the groups (*p* = 0.115). Additionally, no statistically significant differences were observed for CD4^+^ lymphocyte count, number of cigarettes smoked per week, frequency of drinking alcohol per week, ethnicity, marriage, educational, cough, chest imaging results and underlying diseases (*p* > 0.05) in Table 4. Variable assignment was shown in Table 5.

**Table 4:** Analysis of demographic and clinical characteristics of HIV/AIDS and MTB patients.

Variables	Levels	Group		*χ* ^2^	*P*-value
		HIV/AIDS (*n* = 70)	MTB (*n* = 35)		
CD4^+^T lymphocyte count	≥200	59 (84.3)	23 (65.7)	3.681	0.055
	<200	11 (15.7)	12 (34.3)		
Number of cigarettes smoked per week		0 (0,0)	0 (0,0)		0.417^a^
Frequency of drinking alcohol per week		0 (0,10)	0 (0,12)		0.942^a^
Ethnicity	Han	38 (54.3)	18 (51.4)	0.076	0.782
	Zhuang	32 (45.7)	17 (48.6)		
Marriage	Married	48 (68.6)	21 (60.0)		0.634^b^
	Unmarried	14 (20.0)	10 (28.6)		
	Divorced	6 (8.6)	2 (5.7)		
	Widowed	2 (2.9)	2 (5.7)		
Education	Illiterate person	2 (2.9)	1 (2.9)		0.497^b^
	Elementary school	25 (35.7)	15 (42.9)		
	Secondary school	35 (50.0)	14 (40.0)		
	High school	3 (4.3)	4 (11.4)		
	University	5 (7.1)	1 (2.9)		
Occupation	Other*	19 (27.1)	4 (11.4)	5.933	0.115
	Housework and unemployment	7 (10.0)	8 (22.9)		
	Worker	9 (12.9)	3 (8.6)		
	Farmer	35 (50.0)	20 (57.1)		
Cough	No	66 (94.3)	32 (91.4)		0.684^b^
	Yes	4 (5.7)	3 (8.6)		
Results of chest imaging	Normal	45 (64.3)	14 (40.0)	5.591	0.061
	Pulmonary infection	12 (17.1)	10 (28.6)		
	Other**	13 (18.6)	11 (31.4)		
Underlying disease	No	58 (82.9)	25 (71.4)	1.840	0.175
	Yes	12 (17.1)	10 (28.6)		

Except for the number of cigarettes smoked per week and frequency of drinking alcohol per week were described by “median (quartile) [*M* (P_25_, P_75_)],” other data were described by “number of people (proportion/%) [*n* (%)]”; ^a^Wilcoxon rank sum test method; ^b^Fisher exact probability method.

Other*: Business services, officials and staff, driver, retirees and former employees, unspecified.

Other**: Lung lesion, pulmonary nodules, emphysema, tuberculosis, etc.

**Table 5:** Logistic regression analysis variable assignment table.

Variables	Assignment
Groups	HIV/AIDS = 0, MTB = 1
CD4^+^ lymphocyte	≥200 = 1, <200 = 2
Ethnicity	Han = 1, Zhuang = 2
Education	Illiterate person = 1, Elementary school = 2, Secondary school = 3, High school = 4, University = 5
Occupation	Other = 1, Housework and Unemployment = 2, Worker = 3, Farmer = 4
Marriage	Married = 1, Unmarried = 2, Divorced = 3, Widowed = 4
Cough	No = 1, Yes = 2
Results of chest imaging	Normal = 1, Pulmonary infection = 2, Other = 3
Underlying disease*	No = 1, Yes = 2

Underlying disease*: Pulmonary infection, pulmonary heart disease, hepatitis, diabetes, silicosis, chronic bronchitis, hypertension, coronary heart disease, tumor, mental illness, etc.

**Figure 4:**
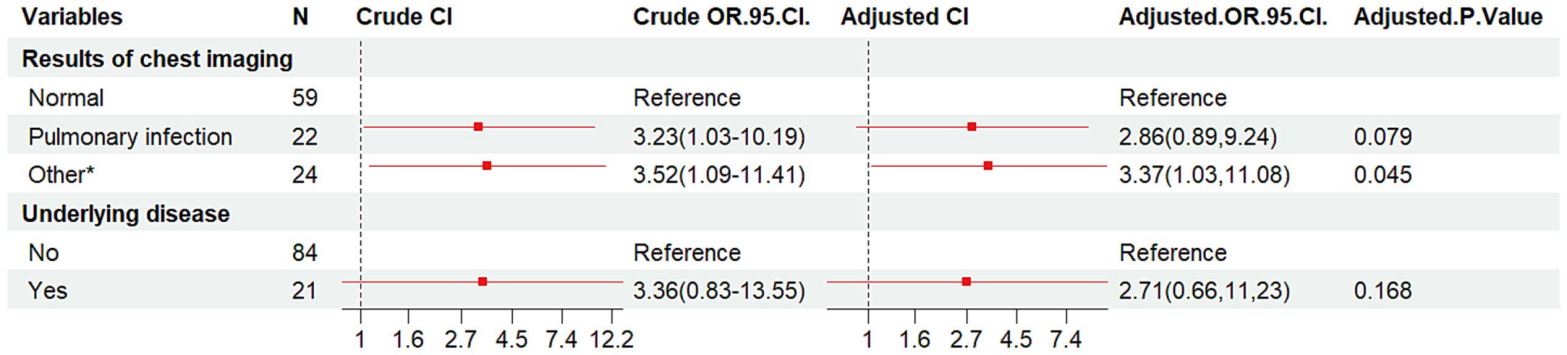
Forest plot of the results of conditional logistic regression analysis of pooled MTB infections. OR, odds ratio; CI, confidence interval; Other*, lung lesion, pulmonary nodules, emphysema, tuberculosis, etc.

The HIV/AIDS group and co-infected with MTB group were used as dependent variables, and 10 factors as independent variable. The results (Table 6 and Figure 4) indicated that compared to individuals with normal chest imaging findings, those with other pulmonary symptoms had an increased risk of MTB co-infection (Crude OR: 3.52, 95% CI: 1.09–11.41; Adj. OR: 3.37, 95% CI: 1.03–11.08).

**Table 6:** Multivariate logistic regression analysis of clinical characteristics of HIV/AIDS group and co-infected with MTB group.

Variables	Crude OR (95%CI)	Adj. OR (95%CI)	*P*-value	*P*-value
			(Wald’s test)	(LR-test)
Results of chest imaging				0.03
Normal (reference)				
Pulmonary infection	3.23(1.03,10.19)	2.86(0.89,9.24)	0.079	
Other*	3.52(1.09,11.41)	3.37(1.03,11.08)	0.045	
Underlying disease				
No (reference)				
Yes	3.36(0.83,13.55)	2.71(0.66,11,23)	0.168	

OR, odds ratio; CI, confidence interval.

Other*: Lung lesion, pulmonary nodules, emphysema, tuberculosis, etc.

**Figure 5:**
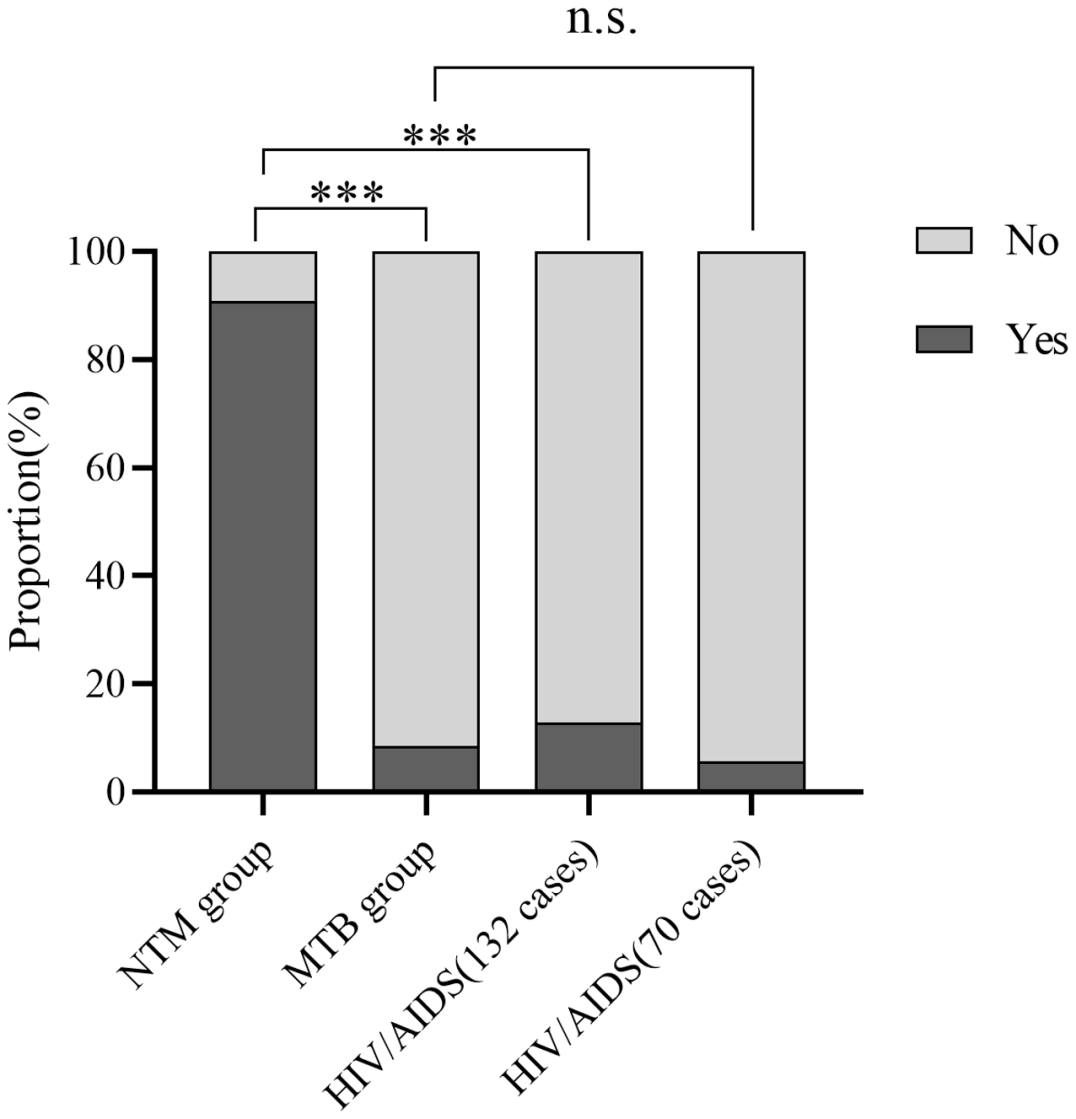
Bar charts displaying the percentage distribution of cough variables across three groups. ***p < 0.001 n.s., not significant.

Cough variables were compared between the NTM and MTB groups, revealing significant differences (*χ*^2^ = 66.070, *p* < 0.001). Univariate analysis showed that cough variables in the NTM group differed significantly from those in the HIV/AIDS group (132 cases, *χ*^2^ = 109.469, *p* < 0.001, Figure 5), while no significant difference was observed between the MTB and HIV/AIDS groups (70 cases, *p* = 0.684).

### 3.4 NTM drug susceptibility test results

Sixty-six NTM strains were tested for resistance to 10 common antibiotics. Resistance rates were PAS 95.5%, INH 93.9%, K 62.1%, EMB 62.1%, LFX 62.1%, CPM 54.6%, RFP 53.0%, MFX 50.0%, AM 50.0%, TH1321 25.8%. Nine drugs had resistance rates over 50.0%, with PAS and INH exceeding 90.0%. TH1321 showed the lowest resistance rate at 25.8% (17/66) (Table 7).

**Table 7:** Resistance rates of NTM strains to 10 commonly used antibiotics detected.

Drug	Resistance	Sensitivity	Drug resistance rate (%)
PAS	63	3	95.5
INH	62	4	93.9
LFX	41	25	62.1
K	41	25	62.1
EMB	41	25	62.1
CPM	36	30	54.6
RFP	35	31	53.0
MFX	33	33	50.0
AM	33	33	50.0
TH1321	17	49	25.8

PAS, para-aminosalicylic acid; INH, isoniazid; LFX, levofloxacin; K, kanamycin; EMB, ethambutol; CPM, capreomycin; RFP, rifampin; MFX, moxifloxacin; AM, amikacin; TH1321, protionamide.

### 3.5 IGRA results and changes in CD4^+^T lymphocyte results

**Figure 6:**
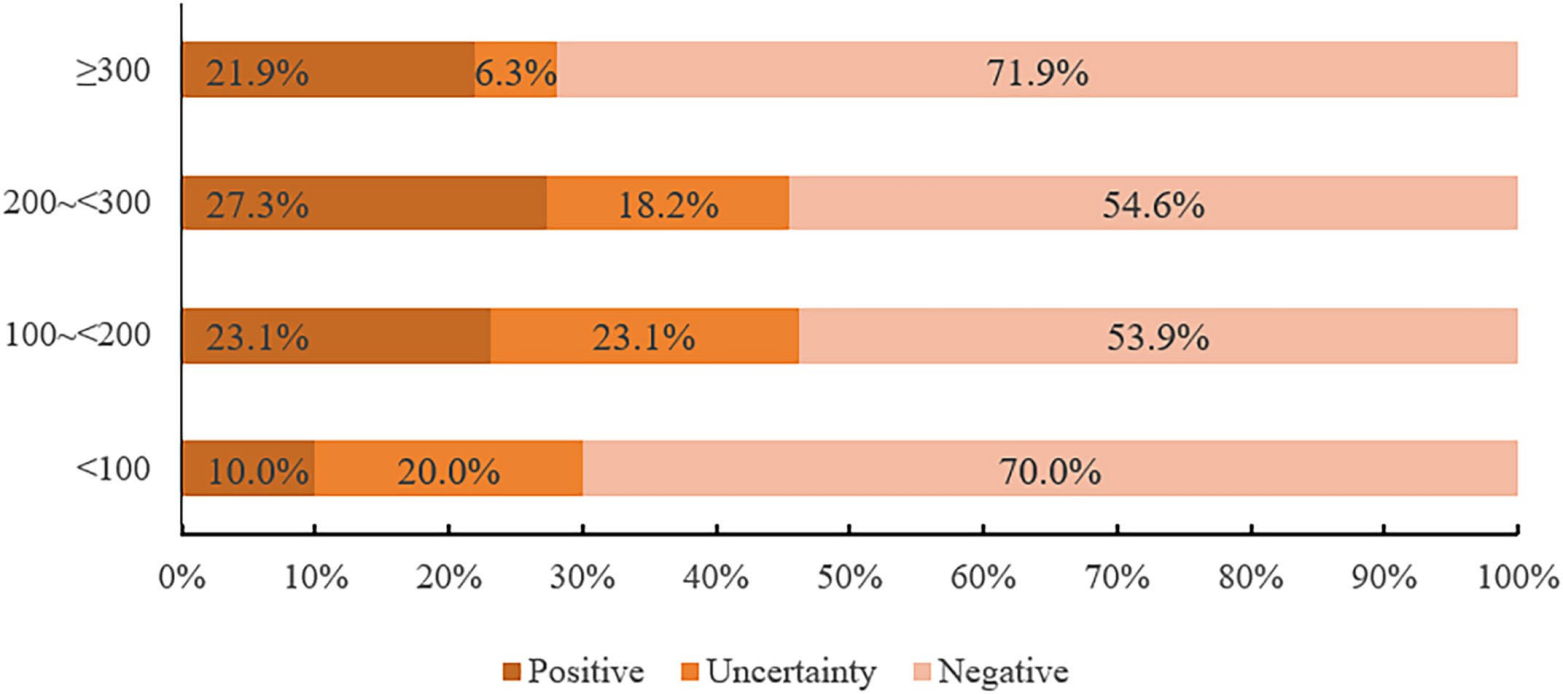
Bar chart of the composition ratio of CD4 + T lymphocyte count and IGRA results.

In this study, 14 cases were IGRA positive, including 6 *M. fortuitum*, 3 *M. avium*, 2 *M. chelonae/abscessus complex*, 1 *M. kansasii*, 1 *M. terrae* and 1 *M. scrofulaceu*. There was no significant difference in the positive rates of the six strains (*p* = 0.123). Among 66 patients, IGRA positive rates were 10.0, 23.1, 27.3, and 21.9% for CD4^+^T lymphocyte counts of 0 ~ <100/mm^3^, 100 ~ <200/mm^3^, 200 ~ <300/mm^3^, and ≥300/mm^3^, respectively. Chi-square tests showed no statistically significant differences between CD4^+^ T cell count and IGRA positive/indeterminate results (*p* = 0.574, *p* = 0.806, *p* = 0.261). These findings were summarized in Figure 6 and Tables 8, 9.

**Table 8:** Relationship between CD4^+^T lymphocyte count and IGRA positive results in patients.

Count of CD4 cell/(n ·mm^−3^)	Cases	Positive	Positive rate/%
0 ~ <100	10	1	10.0
100 ~ <200	13	3	23.1
200 ~ <300	11	3	27.3
≥300	32	7	21.9
Total	66	14	21.2

**Table 9:** Relationship between CD4^+^T lymphocyte count and IGRA indeterminate results in patients.

Count of CD4 cell/(n ·mm^−3^)	Cases	Uncertainty	Uncertainty rate/%
0 ~ <100	10	2	20.0
100 ~ <200	13	3	23.1
200 ~ <300	11	2	18.2
≥300	32	2	6.3
Total	66	9	13.6

## 4 Discussion

In this study, *M. avium*, *M. fortuitum* and *M. chelonae/abscessus complex* were the main pathogens of NTM, accounting for 66.67% (44/66), which was similar to the results of Lu and He (20). However, our results showed a relatively high detection rate of *M. fortuitum* and more common SGM, indicating regional differences in mycobacterial infections, for instance, *Mycobacterium avium-intracellulare complex (MAC)* and *M. kansasii* predominated in Beijing, whereas *M. chelonae/abscessus complex* was more prevalent in Shanghai and Guangzhou (21).

We found that patients in the NTM group had cough symptoms, which was similar to the results of Han et al. (22). Most patients were farmers with low education levels (commonly primary or secondary school), farmers are more prone to NTM infection compared to people in other occupations. The increased risk of NTM infection was associated with advancing age and residing in rural areas (23), similar to the results of our multivariate analysis. Moreover, HIV infection itself causes a significant decline in immune function. Additionally, the combination of being HIV/AIDS patients, experiencing a cough, and farming further elevates the risk of NTM infection, this may be related to poor rural environment and limited medical resources (24). It was also suggested that clinicians to strengthen the prevention and control of NTM in these areas, such as paying attention to the clinical symptoms of high-risk groups, improving the hygiene conditions of domestic water, and popularizing knowledge related to NTM through community health education, so as to reduce the risk of NTM infection. Our analysis showed a lower risk of pulmonary infection in NTM co-infected patients, possibly because NTM infections lack typical lesions and are often misdiagnosed as PTB. In MTB co-infected patients, most were farmers and did not exhibit cough symptoms. Multivariate analysis revealed that chest imaging findings classified as other pulmonary symptoms were linked to an increased MTB infection risk, contrasting with NTM infection conclusions. Further analysis of cough variables showed significant differences in cough prevalence between NTM and MTB groups. Overall, cough symptoms were not prominent in MTB co-infected patients but were associated with a higher NTM infection risk in HIV/AIDS patients. A meta-analysis demonstrated that 78.9% of patients with HIV-associated tuberculosis exhibited at least one symptom, including cough, fever, night sweats, and weight loss (25). Therefore, for HIV/AIDS patients with cough symptoms, it is recommended that while conducting tests for MTB, tests related to NTM should also be carried out to rule out the possibility of NTM infection. NTM co-infected patients often present clinical symptoms (e.g., low-grade fever, cough, chest pain) and imaging changes similar to PTB (26). NTM infections are often misdiagnosed as PTB, resulting in treatment with conventional anti-TB drugs. Due to NTM’s high resistance to these drugs, the treatment outcomes are usually unsatisfactory (27). Data showed that about 1 in 15 patients with suspected PTB in China was infected with NTM. The prevalence of NTM across the country, showing a gradual increase from north to south and from west to east (28). Meanwhile, physicians in TB-endemic areas should carefully distinguish NTM infections from TB. Given the high drug resistance and consistent isolation rates, accurate diagnosis and targeted treatment are crucial (29). Therefore, for patients who lack sufficient evidence for TB diagnosis and have poor response to anti-TB treatment, timely screening for NTM infection should be performed.

The drug resistance of NTM is characterized by a wide range of drug resistance spectrum, and studies have also reported that NTM strains are generally resistant to first-line anti-TB drugs, this may be attributed to drug target gene mutations, the presence of the cell wall barrier, and the efflux pump system (30). We found that HIV/AIDS patients with NTM showed varying degrees of resistance to anti-TB drugs, with a relatively low resistance rate to TH1321 (25.76%). TH1321 may be a candidate drug for NTM treatment. When MTB is resistant to INH, Ethionamide (ETH)/TH1321 is commonly used and is one of the WHO-recommended drugs for multidrug-resistant tuberculosis (31, 32). The structure of TH1321 is analogous to that of INH. It specifically targets the enoyl-CoA carrier protein reductase, which is encoded by the inhA gene, thereby inhibiting mycolic acid synthesis in mycobacteria and compromising the integrity of the cell wall, thereby exerting its anti-TB effect (33). TH1321 is activated by flavin adenine dinucleotide monooxygenase (EthA) to form the TH1321-NAD complex that acts on enoyl-ACP reductase (InhA). In addition, the synthesis of monounsaturated acyl ACP (acyl-ACP) to acyl ACP is reduced, which interferes with the formation of fatty acid synthase II (FASII), blocks the biosynthesis of mycolic acid, and eventually leads to the death of the mycobacteria (34, 35). Our results showed that 17 NTM strains were resistant to TH1321, including 7 *M. avium*, 6 *M. chelonae/abscessus complex*, 2 *M. flavescens*, 1 *M. kansasii* and 1 *M. fortuitum.* The resistance rate to *M. kansasii* was 5.9% (1/17), which was similar to the results of Zhu et al. (36), suggesting TH1321 may be a candidate drug for the treatment of *M. kansasii* infection. The treatment principle of NTM disease is that if there is a strain identification result, the sensitive drug should be selected according to the strain. If the treatment outcome is suboptimal, drug susceptibility testing can be performed (37). Accurate bacterial identification is crucial for patients unresponsive to conventional treatment, with recurrent infections, or immunocompromised. Early strain identification aids in selecting effective drugs, reducing morbidity and mortality (38). NTM strains exhibit varied drug sensitivities and high resistance to anti-TB drugs. Therefore, identifying strains, conducting drug sensitivity tests, and monitoring NTM drug resistance improves clinical treatment guidance.

It has been reported that when the CD4^+^T cells of HIV-infected individuals dropped below 50 cells/μL, they were more likely to develop NTM infections (39). There was a significant correlation between NTM infection and CD4^+^T cell count in AIDS patients. The NTM detection rate increased as CD4^+^T cell count decreased, particularly when CD4^+^ levels fell below 200 cells/μL, significantly raising the risk of NTM infection (40), this differed from our results, which showed no significant difference, possibly due to the relatively small sample size. Mutations in the IFN-γ pathway were associated with an increased risk of infection by strains such as *M. avium* and *M. abscessus* (41). Studies show that IGRA detects IFN-γ from T cells stimulated by MTB-specific antigens ESAT-6 and CFP-10 to determine MTB infection. These antigens are in MTB’s RD1 region. Several NTM type, including *M. kansasii*, *M. marinum*, *M. szulgai*, and *M. gordonae*, encode both specific antigens. Consequently, IGRA may yield a positive result if the body is infected with these NTM species (42, 43). If NTM lacks the two tuberculosis-specific antigens, IGRA results are negative, serving as an early characteristic to differentiate NTM pulmonary disease from PTB (44). In this study, 14 patients (21.2%, 14/66) had the “positive” IGRA results. The overall positive rate and uncertainty rate of IGRA results in NTM-infected patients in the meta-analysis were 16 and 5% respectively, which were higher than those in the present study (45). In our study, six types of IGRA-positive strains were detected, therefore, the IGRA test was of limited value in differentiating TB from NTM diseases (46). Whether the NTM expresses ESAT-6/CFP-10 is the main reason for IGRA positive results, although patients infected with NTM (expressing ESAT-6/CFP-10) can produce IGRA positive results, the positive rate is much lower than that of TB caused by MTB (about 85%), indicating that the diagnostic rate of IGRA is insufficient for diagnosing the infection of NTM species (expressing ESAT-6/CFP-10) (45, 47, 48). In this study, one case of *M. kansasii* was IGRA positive, which may cause IGRA to produce positive results. Many factors can regulate the sensitivity of IGRA, including HIV co-infection, immunosuppression, advanced age (49, 50). The positive result of IGRA may also depend on asymptomatic latent infection in the past (51), for patients with MTB latent infection or a history of TB, detecting NTM in sputum samples alongside a positive IGRA result does not necessarily indicate IGRA positivity is due to NTM. Accurate interpretation requires a comprehensive evaluation integrating medical history, imaging findings, and other clinical data (52). In our study, blood samples were collected from HIV/AIDS patients, suggesting that the collected strains may have the ability to express ESAT-6/CFP-10 antigens, co-infection with MTB, which may lead to the high IGRA positive rate.

There are certain limitations in our study. NTM epidemic strains and drug resistance were analyzed in only 5 cities in Guangxi, with a small sample size for strain identification, limiting the generalizability and extrapolation of the findings. Future studies should expand the scope and sample size in Guangxi to improve NTM strain identification and drug resistance analysis. Additionally, subspecies analysis (e.g., *M. avium*) was not performed, as drug sensitivity varies among subspecies. Further molecular identification using DNA sequencing is recommended. Although this study found that coughing might be a predictive factor for NTM infection in people with AIDS, the sample size was small, which might lead to a type I error in statistics. Further validation through larger sample sizes and cohort studies is needed in the future.

## 5 Conclusion

For HIV/AIDS patients presenting with cough symptoms, it is recommended that molecular biology techniques be employed concurrently with MTB testing to screen for and identify NTM, thereby clarifying the specific type of mycobacterial infection present. IGRA cannot completely distinguish MTB from NTM, and more auxiliary examinations are needed.

## Data Availability

The original contributions presented in the study are included in the article/Supplementary material, further inquiries can be directed to the corresponding author.

## Glossary

AM: Amikacin
CI: confidence interval
CPM: Capreomycin
EMB: Ethambutol
HIV: human immunodeficiency virus;
IFN: interferon
IGRA: Interferon-γ release assay
INH: Isoniazid
K: Kanamycin
LFX: Levofloxacin
MFX: Moxifloxacin
MTB: *Mycobacterium tuberculosis*
NTM: Non-tuberculous Mycobacteria
OR: odds ratio
PAS: Para-aminosalicylic acid
PSM: propensity score matching
PTB: pulmonary tuberculosis
RGM: rapidly-growing mycobacteria
RFP: Rifampin
SGM: slow-growing mycobacteria
TH1321: Protionamide
TB: tuberculosis
