# Supplementary material for: Risk factors and drug resistance of non-tuberculous mycobacteria in HIV/AIDS patients: a retrospective study in southern China
Source: Front Public Health. 2025 Sep 26;13:1660472. doi: 10.3389/fpubh.2025.1660472 (PMC12510969; doi:10.3389/fpubh.2025.1660472)
Supplement: Supplementary file 1 [file Table_1.DOCX]

**2.2 Detection Methods**

**2.2.1 Interferon-γ** **release assay** (**IGRA**) IGRA detection was performed in two steps. First, whole blood was collected, stimulated, and the supernatant harvested. One milliliter of blood was added to each tube, mixed thoroughly, and incubated at 37 ℃ for 16~24 hours. The supernatant was collected after centrifugation. Second, IFN-γ levels were measured using ELISA. The IFN-γ response was calculated as TB antigen tube reading minus negative control tube reading. Results were interpreted as follows: Positive if ≥ 0.35 IU/mL and > 25% above the negative control; Negative if < 0.35 IU/mL or ≥ 0.35 IU/mL but < 25% above the negative control and ≥ 0.5 IU/mL of the positive control; Inconclusive if < 0.35 IU/mL or ≥ 0.35 IU/mL but < 25% above the negative control, < 0.5 IU/mL of the positive control, or > 8.0 IU/mL of the negative control.

**2.2.2** **CD4+ T lymphocyte detection** CD4+ T lymphocytes were detected by flow cytometry using a Facscalibur four-color fluorescent antibody labeling system. 2~3 mL of venous blood was collected into K3EDTA-anticoagulant tubes, mixed well, and stored at 20~25 ℃. For each TrouCOUNT tube, 20 μL of reagent and 50 μL of sample (added by reverse suction) were mixed gently and incubated in the dark at room temperature for 15 minutes. Then, 450 μL of 1×FACS lysate was added, mixed gently, and incubated in the dark at room temperature for an additional 15 minutes until red blood cells were completely lysed. Samples were analyzed using the MultiSET software, which automatically calculated the results.

**2.2.3** **Mycobacterial culture** We used the conventional solid culture method. Using a sterile pipette, we transferred 1~2 mL of sputum into a 50 mL sterile centrifuge tube. Based on the specimen's characteristics, we added 1~2 times its volume of 4% NALC-NaOH digestion solution, tightened the lid, and swirled to liquefy it. After resting at room temperature for 15 minutes for decontamination, we added phosphate buffer (pH 6.8) to the 50 mL mark, mixed thoroughly, and centrifuged at 3500 ×g for 15 minutes. The supernatant was discarded, and 1 mL of phosphate buffer was added and shaken to mix. Growth was negative after 8 weeks of culture.

**2.2.4** **Gene chip identification** Nucleic acid extraction: The strain was scraped, dissolved in sterilized water, inactivated at 95 ℃ for 20 minutes, centrifuged at 12,000 r/min for 5 minutes, mixed with 100 μl water, ultrasonicated for 15 minutes, centrifuged again, and the supernatant was collected (stored at -20 ℃ if needed)；Nucleic acid amplification: Two microliters of DNA template were added to 18 μl reaction mix; amplification was performed: 37 ℃ for 10 minutes, 94 ℃ for 10 minutes, 45 cycles of 94 ℃/30 s, 60 ℃/30 s, 70 ℃/40 s, then 20 cycles of 94 ℃/30 s, 60 ℃/30 s, 72 ℃/1 minute, followed by extension at 72 ℃ for 7 minutes；Hybridization: The mixture was denatured at 95 ℃ for 5 minutes, cooled on ice for 3 minutes, transferred 13.5 μl to the chip lattice, and hybridized at 50 ℃ with rotation for 2 hours. The chip was washed with SDS/SSC, and results were interpreted using a scanner.
